# Contrasting response of fungal versus bacterial residue accumulation within soil aggregates to long-term fertilization

**DOI:** 10.1038/s41598-022-22064-9

**Published:** 2022-10-25

**Authors:** Yingde Xu, Liangjie Sun, Xiaodan Gao, Jingkuan Wang

**Affiliations:** Northeast Key Laboratory of Conservation and Improvement of Cultivated Land, Ministry of Agriculture, College of Land and Environment, Shenyang Agricultural University, No.120 Dongling Road, Shenhe District, Shenyang, 110866 China

**Keywords:** Biogeochemistry, Biogeochemistry, Carbon cycle

## Abstract

Soil microorganisms are critical for soil carbon (C) cycling. They primarily regulate the turnover of the soil organic C (SOC) by adjusting their community structure, and contributing residues with a considerable amount to the resistant SOC. Nevertheless, how long-term fertilization (e.g., the combination of manure and chemical fertilizer) affects the spatial distribution of both living microbial communities and dead microbial residue within soil aggregate fractions remains largely unclear. In this study, we analyzed changes in microbial community (lipid biomarkers) and microbial residue retention (amino sugar biomarkers), and also calculated the contribution of microbial residue to organic C in bulk soil and different soil aggregates (> 2 mm, 1–2 mm, 0.25–1 mm, and < 0.25 mm) in Alfisols treated with 29 years fertilization or no fertilization (control). Our results showed that long-term fertilization significantly increased the mean weight diameter (MWD) of aggregates and organic C contents in all aggregate fractions. The fertilization treatment increased the contents of PLFAs and microbial residue C, but the relative contribution of microbial residue to SOC was higher in the control (56.8% vs. 49.0%), due to the low SOC background caused by much lower level of non-microbially derived C input. These results suggested that long-term fertilization could increase SOC by accumulating both plant- and microbial-derived C, while the C deficient soil is more dependent on the accumulation of microbial residues. Long-term fertilization promoted the enrichment of bacterial-derived muramic acid in micro aggregates, but increased the proportion of fungal-derived glucosamine in macro aggregates. Meanwhile, the contribution of bacterial residue to organic C in the fertilization treatment was higher in micro aggregates (7.6% for > 2 mm vs. 9.2% for < 0.25 mm aggregate), while the contribution of fungal residue was higher in macro aggregate fractions (40.9% for > 2 mm vs. 35.7% for < 0.25 mm aggregate). The above results indicated that long-term fertilization could drive the differentiation of heterogeneous microbial residue accumulation patterns that significantly alter the contribution of fungal- versus bacterial-derived C to organic C within soil aggregate fractions.

## Introduction

Soil organic carbon (SOC) sequestration is critical for agroecosystem sustainability and world food security^1^. Agricultural soil carbon (C) dynamics reflect the long-term balance between inputs of organic materials (e.g., plant residues and manure) and losses of organic C^2^. They are ultimately the consequence of microbial metabolism^3–5^. Soil microorganisms could not only liberate C to the atmosphere via degradation (i.e., catabolism), but also assimilate C through converting net primary production into biomass (i.e., anabolism)^6^. Subsequently, large amounts of microbial residues continuously accumulate within the soil matrix as SOC^7–9^. In this context, the microbial contribution to SOC accumulation is closely related to microbial biomass, community structure, and production^5,10^.

Differentiating the microbe-derived C from bulk SOC has long been a technical challenge^11^. In recent years, emerging studies recognized the essential role of biomarker analysis in indicating how the microbial residue contributed to SOC maintenance^7,11,12^, and how the soil microbial community regulated the microbial residue accumulation^13,14^. Particularly useful biomarkers are phospholipid fatty acids (PLFAs) and amino sugars, the former can serve as indicators for viable microbial biomass and community composition^15–17^, and the latter are sensitive indicators to reveal microbial residue storage at the community level (fungi and bacteria), and to access the contribution of microbial necromass to SOC accrual^18–20^. Linking snapshot variation in PLFAs with the legacy effects of amino sugars may provide more process-based information about microbe-mediated SOC turnover and stabilization^12,13,21^.

Soil microbe-mediated C processes are strongly influenced by agricultural management regimes such as fertilizer application^22,23^. Due to long-term intensive tillage and shortage of organic matter return, significant SOC loss appeared in agroecosystems of Northeastern China^24^. To date, applying organic manure combined with chemical fertilizer is one realistic and effective alternative in agroecosystem to maintain soil fertility and crop productivity^25^. The effects of organic manure and chemical fertilizer input on soil microbial biomass, activity, and community structure have been repeatedly investigated^25–27^. Soils with organic fertilizer can generally maintain high microbial biomass^23,28,29^, but there is no agreement on microbial community structure. The application of manure tended to shift the microbial community structure to a state with higher bacterial richness^30,31^, whereas Heijboer^32^ found that organic manure input had a positive effect on fungal biomarker proportion. Moreover, considering that microbial residue reflects the time-integrated dynamics of microbial community structure^12^, knowledge about the microbial community and its induced dynamics of microbial residue composition (i.e., bacterial *vs*. fungal residue) after long-term fertilization is imperative, which will help elucidate the mechanisms of SOC formation and accumulation.

Furthermore, SOC sequestration comprises a hierarchy of biological processes at the spatial dimension of soil aggregates^33,34^. Soil aggregates can also regulate microbial communities by providing them with diverse micro-niches of various pore spaces and substrate qualities^29,35,36^. Therefore, any changes in soil aggregate composition caused by fertilization may influence C sequestration by affecting microbial community structure and microbial residues. Macro aggregates were formed through binding weathered parent materials with decomposed organic matters (e.g., plant residue)^37^. They have more labile substrates and a higher C turnover rate than micro aggregates^36,38,39^. As a result, more macro aggregates in the soil can promote a great level of microbial activity and increase the fungal biomarker content^10,16^, suggesting a preference of microorganisms for living in different aggregate fractions. Studies also confirmed the accumulation of individual amino sugar largely dependent on the physical protection of the soil aggregate structure^4,10^. However, it is unclear whether the distribution of microbial residues in aggregates is consistent with that of microbial communities. More importantly, impacts of fertilization on soil aggregate composition are fairly well represented^27,40^, but the understanding of how the spatial distribution of microbial community and their residues respond to long-term fertilization is still not enough.

The objective of this study was to elucidate the influence of 29 years of continuous fertilization, the combination of manure and chemical fertilizer, on soil microbial community structure and microbial residues retention within different aggregate fractions. The changes in microbial communities and microbial residues were revealed by PLFA and amino sugar biomarkers, respectively. Our investigations are based on the following hypotheses: (1) given that long-term fertilization can generally promote microbial biomass synthesis^28,29^, microbial residues would accumulate more and contribute more to organic C in aggregate fractions under long-term fertilization; (2) considering that fungi are the main decomposers of exogenous recalcitrant organic matter^41^, long-term fertilization would increase the proportion of fungal PLFA and necromass production; (3) the ratio of fungal/bacterial residues would be greater in macro aggregates than that in micro aggregates.

## Materials and methods

### Study site and soil sampling

The study site is located at the long-term fertilization experimental station (41°49’N, 123°34’E) of Shenyang Agricultural University in Liaoning province, Northeast China. The area exhibits a typical temperate continental monsoon climate, with a mean annual air temperature of 7.6 ºC, and a mean annual precipitation of 705 mm. Precipitation falls mainly during the months from April to September. The soils are classified as Hapli-Udic Alfisol according to the USDA Soil Taxonomy. The field experiment was initiated in the spring of 1987 using a completely random design with three replicates. Each plot was 9.6 m long and 7.2 m wide. Yearly, chemical and organic fertilizers were broadcast as basal dressing in the fertilized field plots before planting. Subsequently, ridges (20 cm high, 60 cm wide) were produced by a ridge plow pulled by a tractor. Maize (*Zea mays* L.) was then sown at 5 cm depth on the ridges at a spacing of 30 cm in early May. About 10 days after planting, herbicides atrazine and acetochlor were sprayed in each plot at the rates of 1.5 kg ha ^−1^ and 6 kg ha^−1^, respectively. None of the treatments were artificially irrigated during the maize growing season, and the water used for crop growth mainly came from precipitation. Maize was harvested manually for grain in early October. The maize yield was estimated according to the grain biomass of the central rows of each plot. The aboveground maize straws were hand-cut with sickle at the connection of the root and stem, and they were removed from the field after the harvest annually.

The treatments selected for the present study were: (1) no fertilization (defined as control); (2) fertilization with organic manure (270 kg N ha^−1^ y^−1^) combined with N (135 kg N ha^−1^ y^−1^) and P_2_O_5_ (67.5 kg P_2_O_5_ ha^−1^ y^−1^) (defined as fertilization treatment). The pig compost was used as organic manure, containing 150 g kg^−1^ total organic C, 10 g kg^−1^ total N (TN), 10 g kg^−1^ P_2_O_5_, and 4 g kg^−1^ K_2_O on a dry weight basis. Urea and ammonium dihydrogen orthophosphate were used as N and P chemical fertilizers.

Five randomized soil samples per plot were collected from the topsoil (0–20 cm) on late April 2016 before maize sowing. The soil samples from the same plot were composited gently. Fresh composite samples were transferred into hard plastic boxes and transported to the laboratory as soon as possible. Once in the laboratory, the soil samples were sieved to pass through a 5-mm sieve by gently breaking soil clods along natural breaking points, and all visible fragments, including roots and debris, were removed by tweezers. Then, one part of each soil sample was sieved in different aggregate fractions after moisture adjustment. The other part was air-dried, ground, and passed through a 0.149-mm mesh for basic property analyses. The organic C and TN were analyzed using an Elemental Analyzer (Elementar, Germany). The soil pH (soil: water, 1:2.5) was measured using a Thunder Magnetic pH Meter (PHS-3B, China). Soil moisture was determined by drying at 105 °C for 8 h. The ammonium N and nitrate N were extracted by 2 M potassium chloride (KCl), and then measured using the AA3 continuous flow analytical system (Auto-Analyzer 3, SEAL, Germany). The available phosphorus (P) in the soil was extracted by 0.5 M sodium bicarbonate (NaHCO_3_) solution (pH = 8.5) and analyzed using the molybdenum blue method; the available potassium (K) was extracted by 1 M ammonium acetate (CH_3_COONH_4_) solution (pH = 7) and measured using the flame photometer. The basic properties of soil samples are presented in Table 1. Besides, we declare that all experimental research and field studies on plants were performed in accordance with the relevant institutional, national, and international guidelines and legislation.

**Table 1:** Basic properties of soil samples.

	SOC	TN	Ammonium N	Nitrate N	Available P	Available K	C/N	Clay	PLFAs	pH	Yield
	(g kg^-1^)	(g kg^-1^)	(mg kg^−1^)	(mg kg^−1^)	(mg kg^−1^)	(mg kg^−1^)		(%)	(nmol g^-1^)	(H_2_O)	(t ha^-1^)
Control	11.2 ± 0.1	1.1 ± 0.1	12.5 ± 0.5	33.0 ± 1.6	13.5 ± 0.4	90.3 ± 2.1	10.2 ± 0.6	25.5 ± 2.4	22.7 ± 1.1	6.0 ± 0.2	3.8 ± 0.3
Fertilization	17.6 ± 0.4	2.2 ± 0.0	13.5 ± 0.4	198.3 ± 4.3	245.0 ± 2.8	228.5 ± 6.2	8.0 ± 0.0	20.0 ± 1.9	30.4 ± 1.5	5.7 ± 0.1	11.0 ± 1.0

The values are shown as means ± standard deviation (*n* = 3).

### Soil aggregate fractionation

Both wet sieving and air drying can damage the in situ links between the aggregates obtained and their indigenous microbial inhabitants^35^. Therefore, we chose to separate aggregates into four size classes (> 2, 1–2, 0.25–1, and < 0.25 mm) through sieving fresh soils according to Helgason^35^ and Wang^29^. Briefly, soil samples were adjusted to 12% gravimetric moisture content before the sieving procedure. The sieving process was carried out with a fractionator (Retsch AS 200, Germany). 200 g fresh soils were sieved using the 2 mm, 1 mm, and 0.25 mm sieves in turn for 15 s, 20 s, and 45 s, respectively. The soils left on the sieves and passed through the last sieve were collected separately. Finally, four aggregates fractions were obtained: > 2 mm (large macro aggregates), 1–2 mm (small macro aggregates), 0.25–1 mm (large micro aggregates), and < 0.25 mm (small micro aggregates, also including sand and silt)^42^. The amount of soils in each aggregate fraction was used to calculate the mean weight diameter (MWD) of aggregates based on the dry weights^43^. After the soil aggregate fraction procedure, one part of soil aggregates was freeze-dried in a vacuum freeze dryer (SCIENTZ-10 N, China) immediately for phospholipid fatty acid (PLFA) analysis. The other part was air-dried, ground, and passed through a 0.149-mm mesh for amino sugar analysis.

### Phospholipid fatty acid analysis

The PLFA extraction and quantification methods were conducted following the procedures of Bossio^44^ as modified by Denef^45^. Briefly, lipids were extracted from 4 g freeze-dried soil in a single-phase chloroform–methanol-citrate buffer (1:2:0.8) on a horizontal shaker (250 rpm) for two and half hours. After centrifugation for 10 min at 4000 rpm, the supernatant was transferred to another glassware tube. Then the remained soil was vortexed for 2 h with an additional volume of the buffer (7.60 mL) and re-extracted. The combined supernatant was added with citrate buffer (16 mL) and chloroform (CHCl_3_) (16 mL), and then left standing overnight to separate into two phases. The CHCl_3_ phase was collected and concentrated under N_2_ at 28 °C. Phospholipids were separated from neutral lipids and glycolipids on a standard solid phase extraction (SPE) tube (6 mL, 500 mg, Supelco, USA). The tube was firstly conditioned with CHCl_3_ (5 mL). The lipids were transferred thoroughly into the SPE tube with CHCl_3_ (3 × 250 μL). Neutral lipids and glycolipids were eluted with chloroform (8 mL) and acetone (16 mL) separately. Phospholipids were obtained from methanol elution (8 mL) and concentrated under N_2_, and were methylated by dissolving in 1:1 methanol-toluene and 0.2 M potassium hydroxide (KOH) solution at 35 °C for 15 min to derivatize them to their respective fatty acid methyl esters (FAMEs). Methyl nonadecanoate fatty acid (19:0, Sigma-Aldrich, USA) was added before quantification as an internal standard. The FAMEs were identified and quantified by an Agilent 7890A GC equipped with MIDI peak identification software (Version 4.5; MIDI, USA). PLFAs 16:0 and 18:0 generally occur in all living cells^46^. PLFAs i15:0, a15:0, i16:0, i17:0 and a17:0 were used as biomarkers for gram-positive bacteria (G +)^47^; 16:1ω7c, 18:1ω7c, cy17:0ω7c, cy19:0ω7c for gram-negative bacteria (G −)^15,48^; 18:2ω6c and 18:1ω9c for saprophytic fungi (SF), and 16:1ω5c for arbuscular mycorrhizal fungi (AMF)^47,49^; 10Me 16:0, 10Me 17:0 and 10Me 18:0 for actinomycetes^48^. In this study, the ratios of different PLFA groups (e.g., G + / G −, AMF/SF, etc.) were used to assess differences in soil microbial communities.

### Amino sugar analysis

Soil amino sugar content was analyzed according to the method of Zhang^50^. Briefly, air-dried soil samples were hydrolyzed with 10 mL 6 M HCl for 8 h at 105 °C. The hydrolysate was filtered through Whatman 2 Qualitative Circles (125 mm diameter), dried at 52 °C using a rotary evaporator, and re-dissolved in deionized water. The pH of the samples was adjusted to 6.6–6.8 and centrifuged (1006 × *g*, 10 min) in 50 mL glass tubes. The supernatant was freeze-dried, after which amino sugars were washed out from the residues with methanol. The recovered amino sugars were transformed into aldononitrile derivatives and extracted with dichloromethane. Excess anhydride was removed with 1 M HCl and deionized water. The amino sugar derivatives were re-dissolved with 200 μL ethyl acetate-hexane (1:1) for final analysis after the removal of dichloromethane under N_2_. Finally, the amino sugar derivatives were separated using an Agilent 7890B gas chromatography (Agilent Technologies, USA) equipped with a HP-5 column (30 m × 0.25 mm × 0.25 μm) and a flame ionization detector. The contents of individual amino sugars, including muramic acid (MurA), glucosamine (GluN), and galactosamine (GalN), were quantified based on the internal standard myo-inositol, which was added before hydrolyzation.

MurA uniquely originates from bacterial peptidoglycan, while GluN is predominantly derived from the chitin of fungal cell walls but can also be found in bacteria and invertebrates^19,51^. The ratio of GluN to MurA was used to evaluate the relative retention of fungal to bacterial residues. GalN can also make a significant contribution to the total amino sugar pool, but its origin is still uncertain^30^. Then, fungal- or bacterial-derived C was estimated according to van Groenigen^52^ and Liang^53^. Fungal GluN was estimated by subtracting bacterial GluN from total GluN, assuming that GluN and MurA occur at a 2:1 M ratio in bacterial cells^54^. Additionally, a factor of 9 was estimated to convert fungal GluN to fungal residue C^19,52,53^. Similarly, a factor of 45 was estimated to convert MurA to bacterial residue C. The calculations were as follows (1–2):

1 $$ {\text{Fungal derived C }} \left( {{\text{mg g}}^{ - 1} } \right) = ({\text{GluN}}\left( {{\text{mg g}}^{ - 1} } \right) \div 179.2 - 2 \times {\text{MurA}}\left( {{\text{mg g}}^{ - 1} } \right) \div 251.2) \times 179.2 \times 9 $$

2 $$ {\text{Bacterial derived C}}\left( {{\text{mg g}}^{ - 1} } \right) = {\text{MurA}}\left( {{\text{mg g}}^{ - 1} } \right) \times 45 $$

where 179.2 and 251.2 are the molecular weight of GluN and MurA, respectively. Total microbial residue C was estimated as the sum of fungal-derived C and bacterial-derived C. The proportion of microbial residue C in SOC represents the microbial residue contribution to SOC sequestration. Besides, the ratios of amino sugars to PLFAs (including GluN/fungal PLFA and MurA/bacterial PLFA) were used to evaluate the apparent efficiency of transformation from living microbial biomass to dead necromass.

### Statistical analysis

Statistical analysis was performed using the software package SPSS 19.0 (IBM, USA). A two-way ANOVA was performed to test the combined effects of fertilization and soil aggregate size on all soil parameters (i.e., soil aggregate composition, organic C, PLFAs, ratios of microbial community composition, amino sugars, GluN/MurA, amino sugars/PLFAs, etc.). The differences in soil parameters between fertilization and no fertilization treatments in the same aggregate fraction were tested using the paired-sample *t-*test. The differences in soil parameters among different soil aggregate fractions in the same fertilization treatment were examined using the one-way ANOVA, followed by Duncan’s test. Significance was reported at *P* < 0.05 level. Principal component analysis (PCA) was used to describe the soil microbial community structure based on the relative abundance of the individual PLFA in the total PLFAs. PCA was carried out with the standard setting in the program CANOCO 4.5. Figures were generated by Origin 8 (Origin Lab, USA).

## Results

### Soil aggregate composition and organic C content

The mass proportion of > 2 mm aggregate and MWD value was significantly higher in the fertilization treatment than that in the control (*P* < 0.05, Table 2). Moreover, the HF treatment significantly increased the organic C contents in all aggregate fractions compared with the control (*P* < 0.05). Organic C contents were generally higher in both large and small micro aggregates (0.25–1 and < 0.25 mm) than in large and small macro aggregates (> 2 and 1–2 mm).

**Table 2:** Soil aggregate composition and organic C content as well as mean weight diameter (MWD).

	Soil aggregate composition (%)				MWD (mm)
	> 2 mm	1–2 mm	0.25–1 mm	< 0.25 mm	
Control	14.1 ± 1.5cB	23.3 ± 0.6bA	55.3 ± 1.1aA	7.3 ± 0.2 dB	1.2 ± 0.1B
Fertilization	24.4 ± 0.2bA	22.1 ± 1.2bA	43.2 ± 2.2aB	10.4 ± 0.3cA	1.5 ± 0.0A

	Organic C content (g kg^–1^ fraction)				
	> 2 mm	1–2 mm	0.25–1 mm	< 0.25 mm	Bulk soil
Control	10.9 ± 0.1bB	10.8 ± 0.3bB	11.2 ± 0.2bB	12.0 ± 0.2aB	11.2 ± 0.1B
Fertilization	17.1 ± 0.1cA	16.8 ± 0.3cA	17.8 ± 0.3bA	18.7 ± 0.1aA	17.6 ± 0.0A

The values are shown as means ± standard deviation (n = 3). Different lowercase letters in a row mean significant difference (*P* < 0.05) among different soil aggregate fractions in the same fertilization treatment, and different uppercase letters in a column mean significant difference (*P* < 0.05) between fertilization and no fertilization treatments in the same aggregate fraction.

### PLFAs and microbial community structure

**Figure 1:**
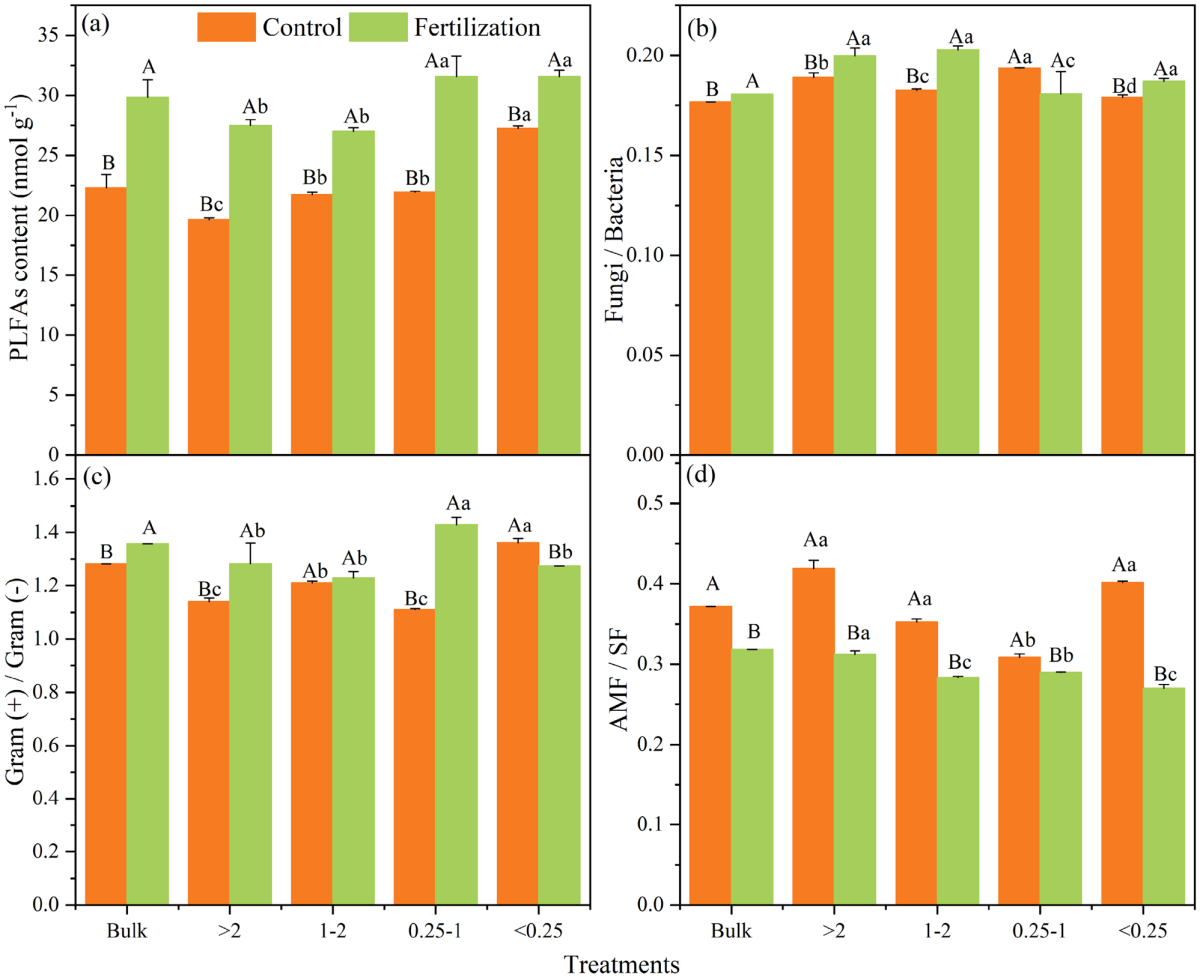
Contents of PLFAs (**a**) and ratios of microbial community composition (**b**–**d**) in bulk soil and different aggregate fractions. Error bars indicate standard deviations. Different lowercase letters mean significant difference (*P* < 0.05) among different soil aggregate fractions, and different uppercase letters mean significant difference (*P* < 0.05) between fertilization and no fertilization treatments.

The fertilization treatment significantly increased the total and individual PLFAs contents in all aggregate fractions compared with the control (*P* < 0.05, Fig. 1a and Table S1). Besides, in bulk soil, the fertilization treatment increased the ratio of fungal- to bacterial PLFA (F/B) and the ratio of G + /G − significantly, but had a negative effect on the AMF/SF ratio (*P* < 0.05; Fig. 1b–d). Moreover, the total PLFA contents were found to increase with decreased aggregate size in a range from 19.6 to 27.5 nmol g^−1^ for the control, and 27.5 to 31.6 nmol g^−1^ for the fertilization treatment (Fig. 1a). The F/B ratio was highest in the 0.25–1 mm aggregate in the control, while it was highest in the macro aggregate in the fertilization treatment (Fig. 1b).

PCA revealed that the fertilization treatment had shifted the microbial community structure to a distinct position from the control (Fig. S1). Specifically, factor loadings of PLFAs suggested that the PLFAs 16:1ω7c, 16:1ω5c, 10Me 16:0, 18:1ω7c, a15:0 and 10Me 18:0 were important in the control, which are considered as indicators of G −, AMF, actinomycete and G + , whereas the PLFAs i16:0, i17:0, a17:0, 18:2ω6c, 18:1ω9c, 10Me 17:0 and cy17:0ω7c were important in the fertilization treatment, which are considered as indicators of G + , SF, G– and actinomycete.

### Amino sugars, the ratio of amino sugars to PLFAs, and microbial residue

**Figure 2:**
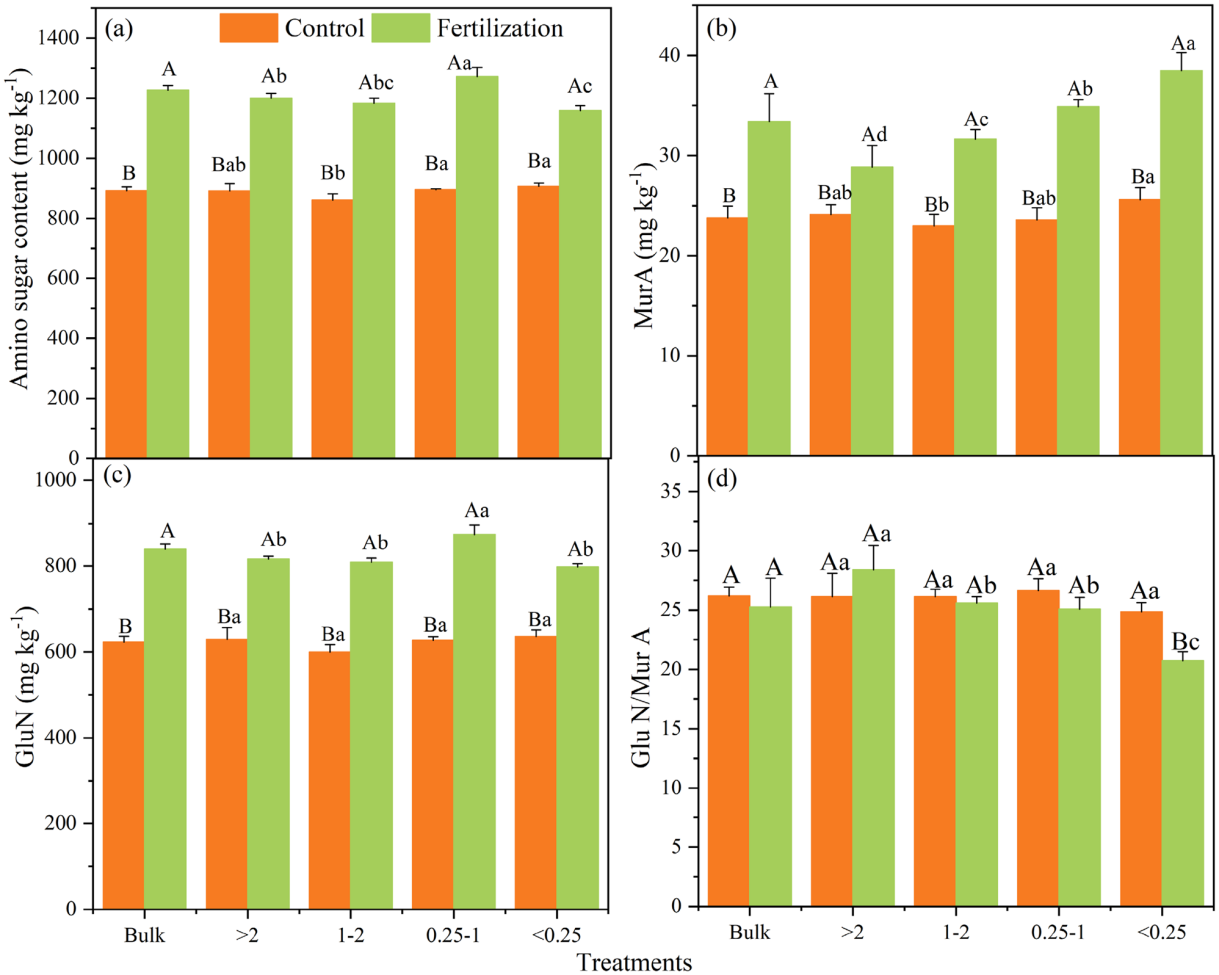
Contents of amino sugars (**a**–**c**) and GluN/MurA ratios (**d**) in bulk soil and different aggregate fractions. Error bars indicate standard deviations. Different lowercase letters mean significant difference (*P* < 0.05) among different soil aggregate fractions, and different uppercase letters mean significant difference (*P* < 0.05) between fertilization and no fertilization treatments.

The amino sugar contents were significantly affected by fertilization, aggregate size, and their interaction (*P* < 0.05, Table 3 and Fig. 2). The total amino sugar contents in the control and fertilization treatments were 893.2 mg kg^−1^ and 1227.1 mg kg^–1^, respectively (Fig. 2a and c). The amino sugar contents were highest in the 0.25–1 mm and < 0.25 mm fraction for control, and highest in the 0.25–1 mm fraction for fertilization treatment (*P* < 0.05). The MurA content increased with the decrease of aggregate size, especially in the fertilization treatment (*P* < 0.05, Fig. 2b). The GluN content did not vary significantly within aggregate fractions in the control (*P* > 0.05, Fig. 2c), while it was highest in the 0.25–1 mm fraction for fertilization treatment (*P* < 0.05). Similarly, aggregate size had no significant effect on GalN content in the control (*P* > 0.05, Table S1), while the GalN contents were highest in 0.25–1 mm and > 2 mm fractions in the fertilization treatment (*P* < 0.05).

**Table 3:** The ANOVA table depicting the effect of fertilization and aggregate size on the dependent variables.

Soil parameters	*P* value		
	Fertilization (F)	Aggregate size (A)	F × A
Soil aggregate composition	< 0.001	< 0.001	< 0.001
Organic C content	< 0.001	< 0.001	0.034
PLFAs content	< 0.001	< 0.001	< 0.001
Fungi/Bacteria	0.001	0.001	< 0.001
Gram(+)/Gram(−)	< 0.001	0.001	< 0.001
AMF/SF	< 0.001	0.001	0.001
Amino sugar content	< 0.001	< 0.001	< 0.001
MurA content	< 0.001	< 0.001	< 0.001
GluN content	< 0.001	0.001	0.002
GluN/MurA	0.065	< 0.001	0.003
Amino sugars/PLFAs	0.020	< 0.001	0.002
The proportion of bacterial residues in SOC	< 0.001	0.204	0.023
The proportion of fungal residues in SOC	< 0.001	< 0.001	0.244
The proportion of microbial residues in SOC	< 0.001	0.083	0.725
Fungal residues/bacterial residues	0.087	< 0.001	0.004

The GluN/MurA ratio showed no significant change after long-term fertilization in bulk soil (Fig. 2d), but within aggregates, the fertilization treatment significantly decreased the GluN/MurA ratio in < 0.25 mm fraction compared to the control (*P* < 0.05). Besides, the GluN/MurA ratio did not change with the variation of soil aggregate size in the control (*P* > 0.05), but it was found to decrease with decreased aggregate size in a range from 28.4 to 20.7 in the fertilization treatment (*P* < 0.05).

**Figure 3:**
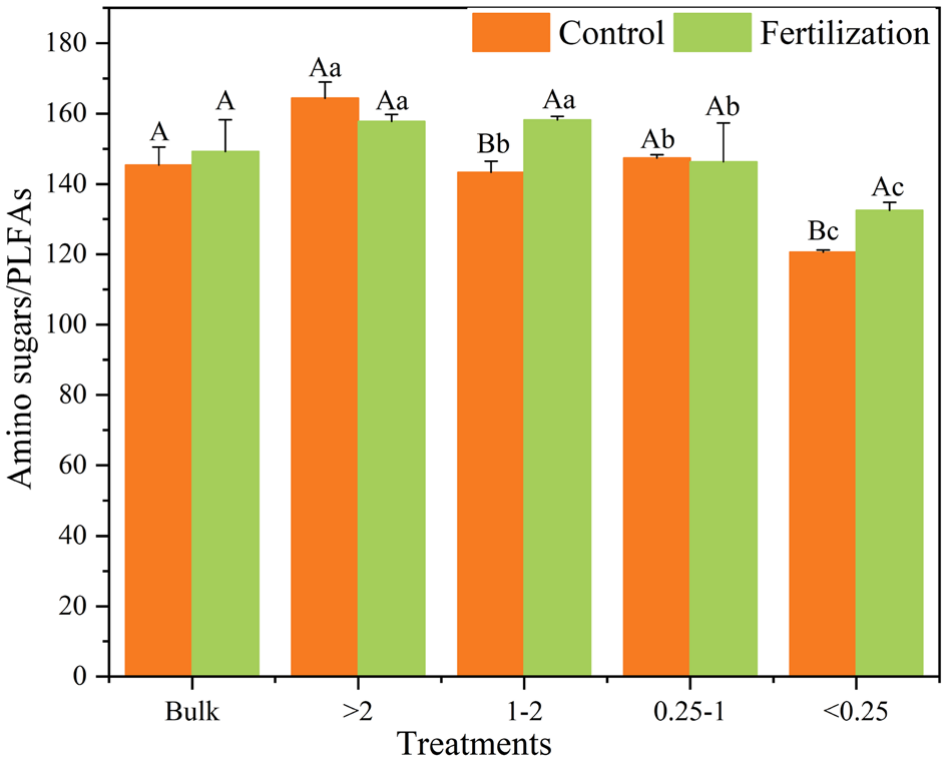
The ratios of amino sugars to PLFAs in bulk soil and different aggregate fractions. Error bars indicate standard deviations. Different lowercase letters mean significant difference (*P* < 0.05) among different soil aggregate fractions, and different uppercase letters mean significant difference (*P* < 0.05) between fertilization and no fertilization treatments.

The amino sugars/PLFAs, MurA/bacterial PLFA, and GluN/fungal PLFA ratios in bulk soil were not affected by fertilization (*P* > 0.05, Figs. 3 and S2). In the control, these three parameters all decreased with the decrease of aggregate size. Long-term fertilization, compared to the control, did not alter the trend of amino sugars/PLFAs and GluN/fungal PLFA ratios within aggregate fractions, but changed the trend of MurA/bacterial PLFA ratio, which showed an increasing trend with the decrease of aggregate size (Fig. S2).

**Figure 4:**
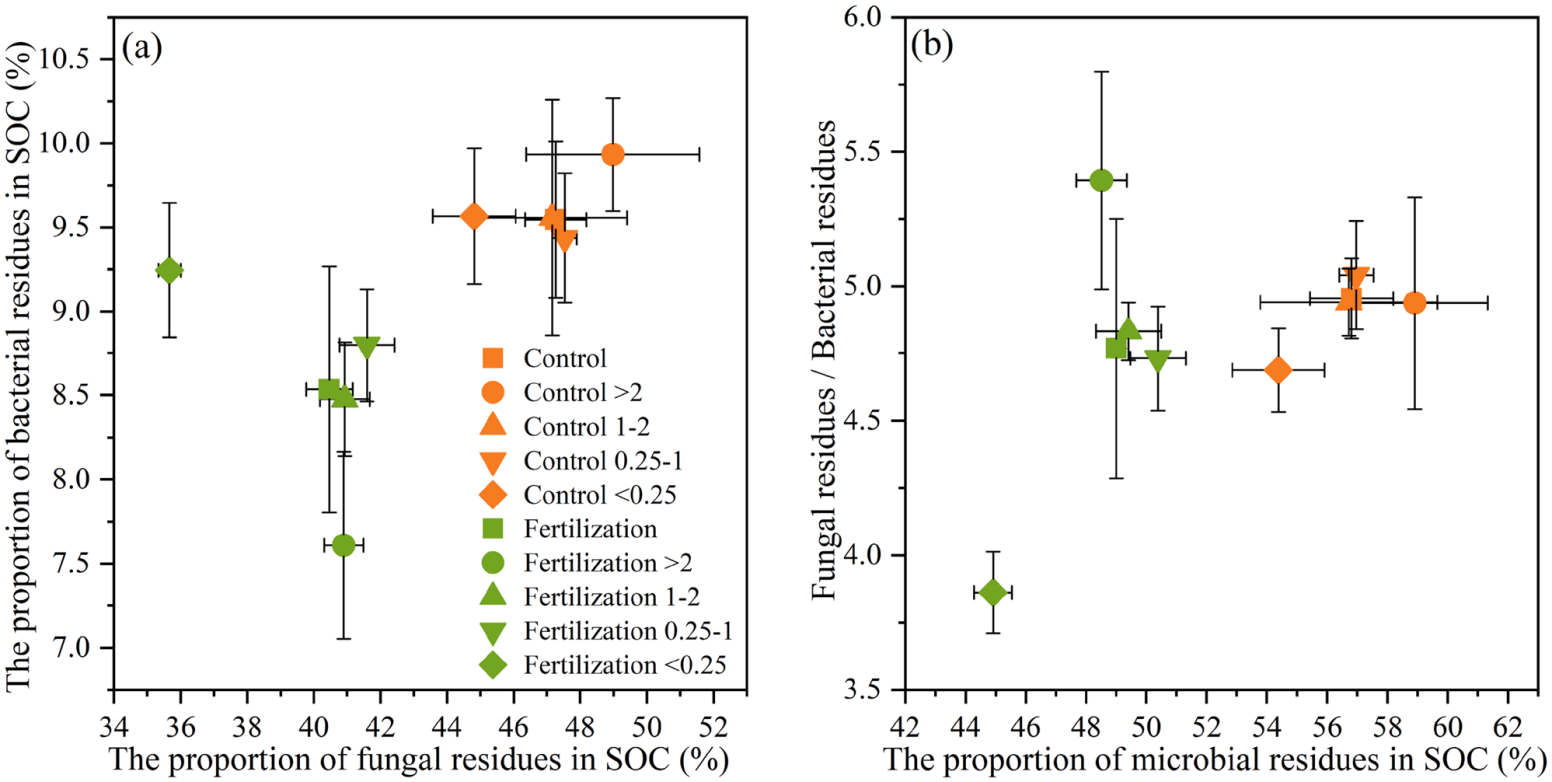
The proportions of bacterial and fungal residues in SOC (**a**), the ratios of fungal residues to bacterial residues, and the proportion of total residues in SOC (**b**) in bulk soil and different aggregate fractions. Error bars indicate standard deviations.

Averaged across the treatments, microbial residues derived 82.9% from fungi and 17.1% from bacteria, and contributed 52.9% of the SOC (Fig. 4). The fertilization treatment caused a significant reduction (13.7%) in the proportion of microbial residues in the SOC, including both fungal residues and bacterial residues. However, the ratio of fungal residues to bacterial residues was not changed by fertilization. In the control, the proportions of fungal residues or bacterial residues in the SOC were not significantly affected by aggregate size (*P* > 0.05). But in the fertilization treatment, the < 0.25 mm fraction had the largest proportion of bacterial residues in the SOC while the lowest proportion of fungal residues in the SOC.

## Discussion

### Dynamics of PLFA and amino sugar biomarkers

In this study, fertilization treatment increased both the PLFA and the amino sugar contents (Figs. 1a and 2a), which supports the first hypothesis that long-term fertilization would promote microbial residue accumulation. The input of organic manure and chemical nutrients provided sufficient available substrate for soil microbial anabolic metabolism^16^, and subsequently, the processes of cell generation, population growth, and cell death were accelerated^55^. Thus, along with the activation of microbial biomass, microbial residues like amino sugars also accumulated in soil microcosms. Meanwhile, long-term fertilization significantly increased the crop yield (Table 1), which in turn delivered more photosynthates (e.g., root debris and exudates) into the soil, also leading to macro aggregate formation, microbial growth, and microbe-derived C deposition^56^.

Soil aggregates with hierarchical compositions and various pore networks provide different habitats for microorganisms^35^. The present study provides evidence that the microbial biomass was greatly affected by aggregate size, with the results showing that the total PLFAs and the biomass of most microbial groups tended to increase with decreasing aggregate size, as did organic C content (Fig. 1a, Tables 1 and Table S1). The present results are inconsistent with those of Six^10^ and Kong^16^, which found that fungi and actinomycetes were more abundant in macro aggregates while bacteria were richer in micro aggregates. Besides, some other studies that did not find any difference in PLFAs abundances among aggregate fractions^35,39^. The discrepancy among studies implies that the variation of microbial biomass could be not only related to the aggregate structure, but also other factors, e.g., the differences in organic C distribution within soil aggregates. Soil aggregate with higher C content can provide more substrates for microbial proliferation, leading to higher microbial biomass^28^.

We found that the contents of amino sugars were highest in the micro aggregates (Fig. 2a). This distribution pattern in aggregates was synchronized with that of living microbial biomass abundance and organic C content, which is consistent with the previous studies^57,58^. According to Liang^59^, soil microbial residues represent a time-integrated index of microbial growth and reproduction. Thus, the dynamics of microbial residues within aggregates would theoretically correlate with living microbial biomass. However, we found this correlation ship was only valid for total amino sugar and MurA (Fig. 2a–b), but not for GluN and GalN (Fig. 2c, Table S2). Therefore, the accumulation of microbial residue should depend not only on the varying biomass of microbial communities, but also on some other factors in many cases^52^. Some studies pointed out that those factors might concern the balance between the production and degradation of microbial anabolic products^10,19^, and mineral or metal oxide content that combined with and fixed the microbial residues^60^. Thus, future research should pay more attention to the various factors that regulate the accumulation of microbial necromass.

GalN is also a critical component of the amino sugars, and it accounted for 28.3% of total amino sugar content in the present study (Table S1). However, the source of GalN is still under debate. The GalN changing trends among aggregate fractions were similar to GluN in our study, which agrees with the finding reported by Engelking^54^ and implies that GalN may have an association with fungi. However, some other studies found a high correlation between GalN and bacterial C^19,30^. Besides, Ding^4^ found that the accumulation pattern of GalN within aggregates differed from that of GluN or MurA. Therefore, the source of GalN still cannot be completely determined.

### Changes in microbial community structure and amino sugar composition

#### Changes in microbial parameters in bulk soil

An apparent change in the microbial community structure caused by long-term fertilization was observed in bulk soil (Fig. 1b–d). Specifically, the AMF/SF ratio was decreased, while the F/B and G + /G − ratios were significantly increased after long-term fertilization, which supports our second hypothesis. Considering that the AMF generally plays a critical role in assistant plant nutrient uptake in a relatively oligotrophic soil environment^61^, the abundant soil nutrients (e.g., N) in the fertilization treatment may decrease the dependency of nutrients from the AMF, and reduce the carbohydrate supply for the mycorrhizal association, resulting in a limitation of in AMF growth and the decrease of AMF/SF ratio^14,62^. However, the increased F/B ratio and decreased AMF/SF ratio imply a favorable condition for saprophytic fungi in the long-term fertilization soil environment. Wang^29^ found that the 23-year manure amendment increased the F/B ratio, which is consistent with our study. The elevated organic substrate and nutrients provided by manure and chemical fertilizers in the fertilization soil eliminate the C and P (the primary limiting nutrient for fungi) limitation, and accelerate the growth of fungi^14^. Besides, large amounts of maize root residues remaining in the fertilization treatment could also contribute to the reproduction of saprophytic populations, because of the predominant role of saprophytic fungi in decomposing cellulose and lignin, the primary components of maize residue^63^. However, it is worthy to mention that, although long-term fertilization increased the F/B ratio, the GluN/MurA ratio did not show significant differences between control and fertilization treatments in bulk soil (Figs. 2d and 3). This is contrary to part of our second hypothesis that the proportion of fungal residues would increase after long-term fertilization. The degradation of fungal residues may be the primary mechanism underlying the asynchronous increase between fungal biomass and fungal residues in the fertilization treatment. Most fungi are obligate aerobes and tend to live in the air-filled spaces in soil^64^, leading fungal residues to be more accessible to microorganisms than bacterial residues^14,64^. Therefore, the decomposition of fungal residues by microorganisms could be promoted by the fertilization treatment due to the increased proportion of macro aggregates. In summary, the narrow variation of Glu/MurA ratio indicates that the composition of accumulated fungal and bacterial residues in the soil could remain in a relatively stable state even with the influence of fertilization.

#### Changes in microbial parameters in soil aggregates

In soil aggregates, the variations of internal micro environmental conditions and organic matter distribution could lead to differentiation of microbial community structure^36^. In the present study, long-term fertilization changed the trend of the F/B ratio within aggregates, i.e., the F/B ratio was highest in the 0.25–1 mm aggregate in the control, while the highest in the fertilization treatment was > 2 mm and 1–2 mm (Fig. 1b). This result indicates that fertilization practice could cause relatively greater enrichment of fungal biomass in the macro aggregates, which could be attributed to the transformation of micro aggregates to macro aggregates^37,65^. The evidence regarding the significantly increased > 2 mm aggregate proportion in the fertilization treatment (Table 2) can support this speculation. Meanwhile, with the increase of available substrates in the fertilization treatment, the organic C content may no longer be an important factor limiting the distribution of fungi in different aggregates, which could be another potential mechanism. Therefore, these results highlight that the changes in microbial community structure are closely related to the alteration of microbial habitat and aggregate turnover induced by long-term fertilization.

Long-term fertilization also made individual amino sugars react differently in aggregate fractions. The MurA has been reported to be stabilized by soil particles via strong ligand exchange and polyvalent cation bridges due to the large amount of charges and strong surface energy^51,66^. Considering that micro aggregates have much higher charges than macro aggregates, MurA was enriched in micro aggregates in our study (Fig. 2). Besides, long-term fertilization further promoted the accumulation of MurA in micro aggregates. This phenomenon mainly resulted in that the GluN/MurA ratio decreased with decreasing aggregate size significantly (Fig. 2d), which seems to be consistent with the trend of F/B in the fertilization treatment, and supports our third hypothesis. We emphasize that although long-term fertilization promoted the combination of micro aggregates into macro aggregates (Table 2), bacterial-derived MurA would also preferentially accumulate and reach saturation first in micro aggregates due to its biodegradability. The above results indicate that there are specific mechanisms for the enrichment of fungal and bacterial residues in different aggregate fractions, and long-term fertilization promotes the accumulation of bacterial residues in micro aggregates relatively to fungal residues.

Moreover, long-term fertilization did not change the trend of amino sugars/PLFAs and GluN/fungal PLFA ratios within aggregate fractions (Figs. 3 and Table S2). However, it significantly increased the ratio of MurA/bacterial PLFA within micro aggregates (Fig. S2), suggesting that fertilization can strongly change the balance between the generation and decomposition of bacterial-derived C. As a result, the retention efficiency of bacterial residue from living biomass to necromass within micro aggregates was improved. This pattern confirms that long-term fertilization plays a vital role in regulating bacterial residue build-up in different aggregates.

However, it is worthy to mention that seasonal change is an important factor affecting the microbial community, as the optimum temperatures of soil fungi and bacteria activities are different^67^. In this study, we only collected soil samples once before planting maize in the spring, which might lead to some uncertainties about soil microbial communities and their residues.

### Microbial residue contribution to SOC sequestration

It has been becoming a consensus that microbial residues could play an important role in forming persistent SOM^4,11,12^. The data presented herein also confirms that the microbial residues comprise a significant fraction of the SOC pool, with a range of 50.0%–56.7% in bulk soil, corresponding well with the previously reported range in agricultural soils^14,57,58^.

Interestingly, the changing patterns of microbial residue percent in SOC showed a distinct picture of amino sugar accumulation. We found that long-term fertilization reduced the contribution of microbial residues to SOC (Fig. 4), though it promoted the amino sugar amount (Fig. 2), which rejects the first hypothesis. In the fertilization treatment, the large amount of organic manure application could supplement the SOC pool and promote plant-derived C input (e.g., crop root biomass and root exudates)^68^, which dilutes the proportion of microbial-derived C in SOC^51^. Moreover, the limited organic matter input in the control may lead to substrate deficiency for microorganism metabolism. Thus, soil microorganisms would utilize more newly added plant-derived C, and consequently convert more substrate into microbial-derived C eventually^69^. Huang^58^ found a low contribution of microbial residues in forest soil with high organic matter inputs from both above- and belowground biomass, which supports our result. These results suggest that long-term fertilization increases SOC through the accumulation of both plant- and microbial-derived C, while the soil with limited C source is more dependent on the accumulation of microbial residues.

Bacterial (or fungal) residues contributed rather similar to the organic C of different aggregate fractions in the control (Fig. 4), but contributed differently after long-term fertilization, i.e., the contribution of bacterial residues to organic C in the fertilization treatment was the highest in < 0.25 mm fraction, and fungal residues was the lowest in < 0.25 mm fraction (Fig. 4). The results indicated that the long-term fertilization drove the differentiation of heterogeneous microbial residues contribution to organic C among aggregates. This alteration possibly results in a potential cascading process of C storage in soil aggregate scale. Specifically, compared with the micro aggregates, the higher proportion of fungal residues in the macro aggregates (Figs. 2 and 4) in the fertilization treatment is more conducive to improving SOM stabilization due to its refractory property and low turnover rate^4^, and also enhances the stability of macro aggregates^65^. In contrast, the enrichment of bacterial residue within micro aggregates in the fertilization treatment is of great importance for C sequestration, since chemical adsorption produced by clay particles protects the easily broken-down bacterial residue from the microbial attack. The above results indicate that long-term fertilization can trade off the microbe-derived organic matters (fungal- *vs*. bacterial residue) in different aggregate fractions to maximize the SOC sequestration and aggregate stability.

## Conclusions

**Figure 5:**
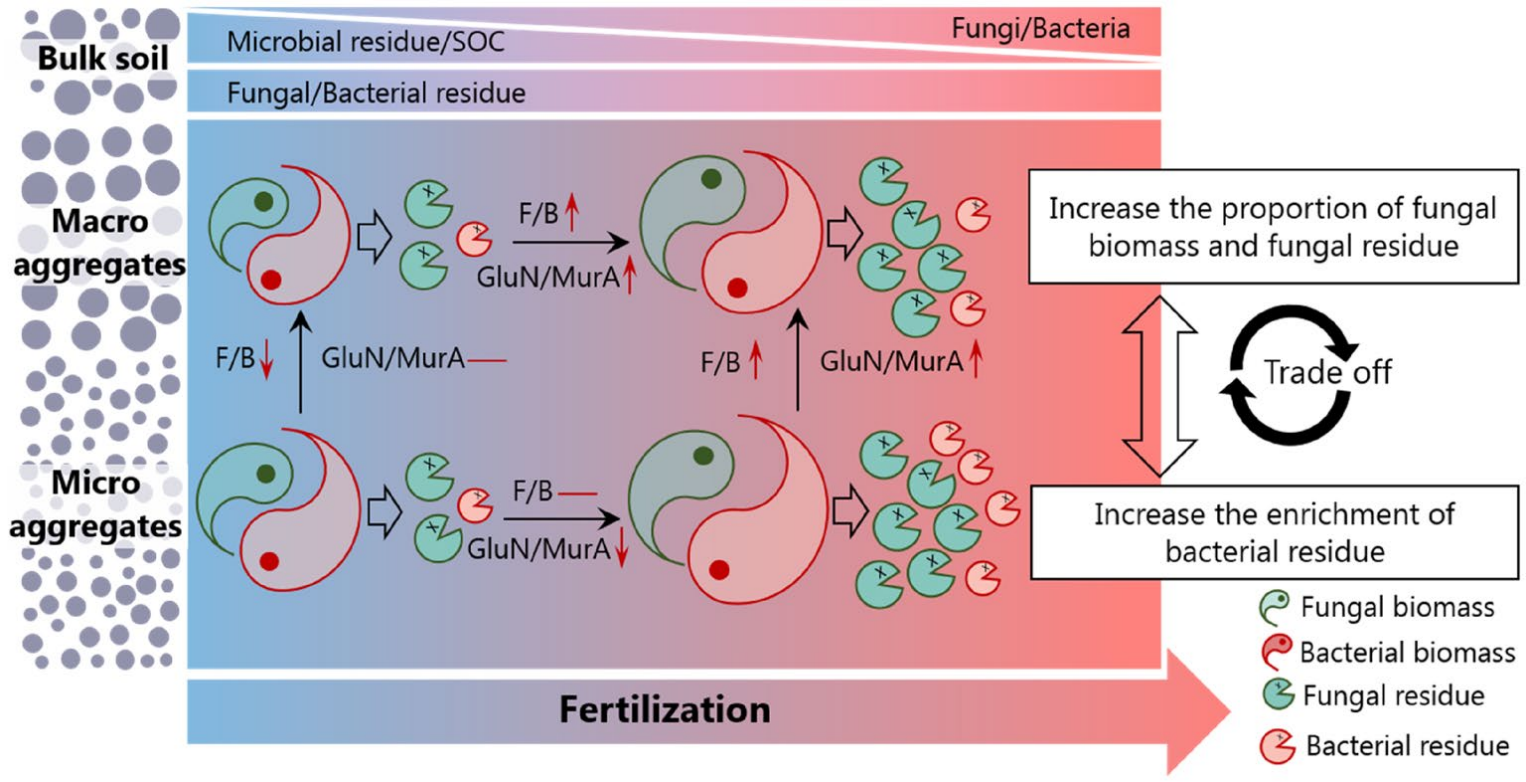
A conceptual diagram illustrating the main effects of long-term fertilization on microbial community and microbial residue accumulation. The short arrows↓and↑represent decrease and increase in corresponding parameter, respectively. SOC, F/B, and GluN/MurA represent soil organic carbon, the ratio of fungal biomass to bacterial biomass, and the ratio of glucosamine to muramic acid, respectively.

We synthesized the present results into a framework (Fig. 5). The results demonstrated that long-term fertilization was efficient in stimulating microbial biomass and residue C levels, while it resulted in a lowered contribution of microbial residues to SOC (49.0% for fertilization treatment *vs*. 56.8% for control). Furthermore, long-term fertilization increased the proportion of fungal biomass and residues in the macro aggregates, while it promoted the accumulation of bacterial residue in micro aggregates. Meanwhile, long-term fertilization drove the differentiation of heterogeneous microbial residues’ contribution to organic C among aggregates. Specifically, the contribution of bacterial residues to organic C in the fertilization treatment was higher in micro aggregates (7.6% for > 2 mm vs. 9.2% for < 0.25 mm aggregate), while the contribution of fungal residues was higher in macro aggregate fractions (40.9% for > 2 mm vs. 35.7% for < 0.25 mm aggregate). Overall, this study has important implications for predictions of the effects of long-term fertilization on SOC dynamics and sequestration controlled by microbial activities in the agroecosystem.

## Supplementary Information

Supplementary Information.

## Data Availability

The datasets used and/or analysed during the current study available from the corresponding author on reasonable request.
