# Supplementary material for: Contrasting response of fungal versus bacterial residue accumulation within soil aggregates to long-term fertilization
Source: Sci Rep. 2022 Oct 25;12:17834. doi: 10.1038/s41598-022-22064-9 (PMC9596480; doi:10.1038/s41598-022-22064-9)
Supplement: Supplementary file 1 — Supplementary Information. [file 41598_2022_22064_MOESM1_ESM.docx]

**Contrasting response of fungal *vs*. bacterial residue accumulation within soil aggregates to long-term fertilization**

Yingde Xu, Liangjie Sun*, Xiaodan Gao, Jingkuan Wang*

Northeast Key Laboratory of Conservation and Improvement of Cultivated Land, Ministry of Agriculture, College of Land and Environment, Shenyang Agricultural University, Shenyang 110866, China

**^*^Corresponding author:**

Liangjie Sun (E-mail address: sunlj@syau.edu.cn; Address: No.120 Dongling Road, Shenhe District, College of Land and Environment, Shenyang Agricultural University, Shenyang, Liaoning 110866, China)

Jingkuan Wang (E-mail address: jkwang@syau.edu.cn; Address: No.120 Dongling Road, Shenhe District, College of Land and Environment, Shenyang Agricultural University, Shenyang, Liaoning 110866, China)

**Supporting tables**

**Table S1** Contents of individual PLFA in bulk soil and different aggregate fractions. The values are shown as means ± standard deviation (n=3). Different lowercase letters in a column mean significant difference (*P* < 0.05) among different soil aggregate fractions in the same fertilization treatment, and * means significant difference (*P* < 0.05) between fertilization and no fertilization treatments in the same aggregate fraction.

| Teratments | Aggregates size | General  (nmol g^−1^) | Gram (+)  (nmol g^−1^) | Gram (–)  (nmol g^−1^) | Fungi  (nmol g^−1^) | AMF  (nmol g^−1^) | Actinomycete  (nmol g^−1^) |
| --- | --- | --- | --- | --- | --- | --- | --- |
| Control | Bulk soil | 4.1±0.2* | 6.7±0.3* | 5.3±0.3* | 2.1±0.1* | 0.8±0.1* | 3.4±0.2* |
|  | >2 mm | 3.3±0.0d* | 5.6±0.0d* | 4.9±0.1d* | 1.8±0.0d* | 0.8±0.1b* | 3.2±0.0c* |
|  | 1–2 mm | 3.8±0.0c* | 6.4±0.1c* | 5.3±0.0c* | 2.1±0.0c* | 0.7±0.0c* | 3.5±0.1b* |
|  | 0.25–1 mm | 3.7±0.1b* | 6.2±0.0b* | 5.5±0.0b* | 2.3±0.0b* | 0.7±0.0d* | 3.6±0.2b* |
|  | <0.25 mm | 5.0±0.2a* | 8.4±0.1a* | 6.1±0.1a* | 2.5±0.0a* | 1.0±0.1a* | 4.3±0.0a* |
| Fertilization | Bulk soil | 5.7±0.3 | 9.2±0.4 | 6.8±0.3 | 3.0±0.1 | 0.9±0.1 | 4.2±0.2 |
|  | >2 mm | 5.0±0.2b | 8.2±0.1c | 6.4±0.3b | 3.0±0.1c | 0.9±0.0a | 3.9±0.2b |
|  | 1–2 mm | 4.9±0.4b | 7.9±0.1c | 6.4±0.1b | 3.0±0.0c | 0.9±0.2c | 3.9±0.1b |
|  | 0.25–1 mm | 6.2±0.8a | 9.7±0.3a | 6.8±0.3ab | 3.2±0.0b | 0.9±0.1a | 4.7±0.4a |
|  | <0.25 mm | 6.2±0.2a | 9.2±0.1b | 7.2±0.1a | 3.3±0.1a | 0.9±0.0b | 4.7±0.1a |

**Table S2** Contents of galactosamine (mg kg^−1^) in bulk soil and different aggregate fractions. The values are shown as means ± standard deviation (n=3). Different lowercase letters in a row mean significant difference (*P* < 0.05) among different soil aggregate fractions in the same fertilization treatment, and * means significant difference (*P* < 0.05) between fertilization and no fertilization treatments in the same aggregate fraction.

| Treatments | >2 mm | 1−2 mm | 0.25−1 mm | <0.25 mm | Bulk soil |
| --- | --- | --- | --- | --- | --- |
| Control | 238.5±9.1a* | 237.8±5.1a* | 246.2±8.0a* | 245.4±4.9a* | 247.0±3.7* |
| Fertilization | 354.7±15.1a | 341.7±9.3ab | 362.5±15.8a | 322.9±8.6b | 354.2±6.8 |

**Supporting figures**


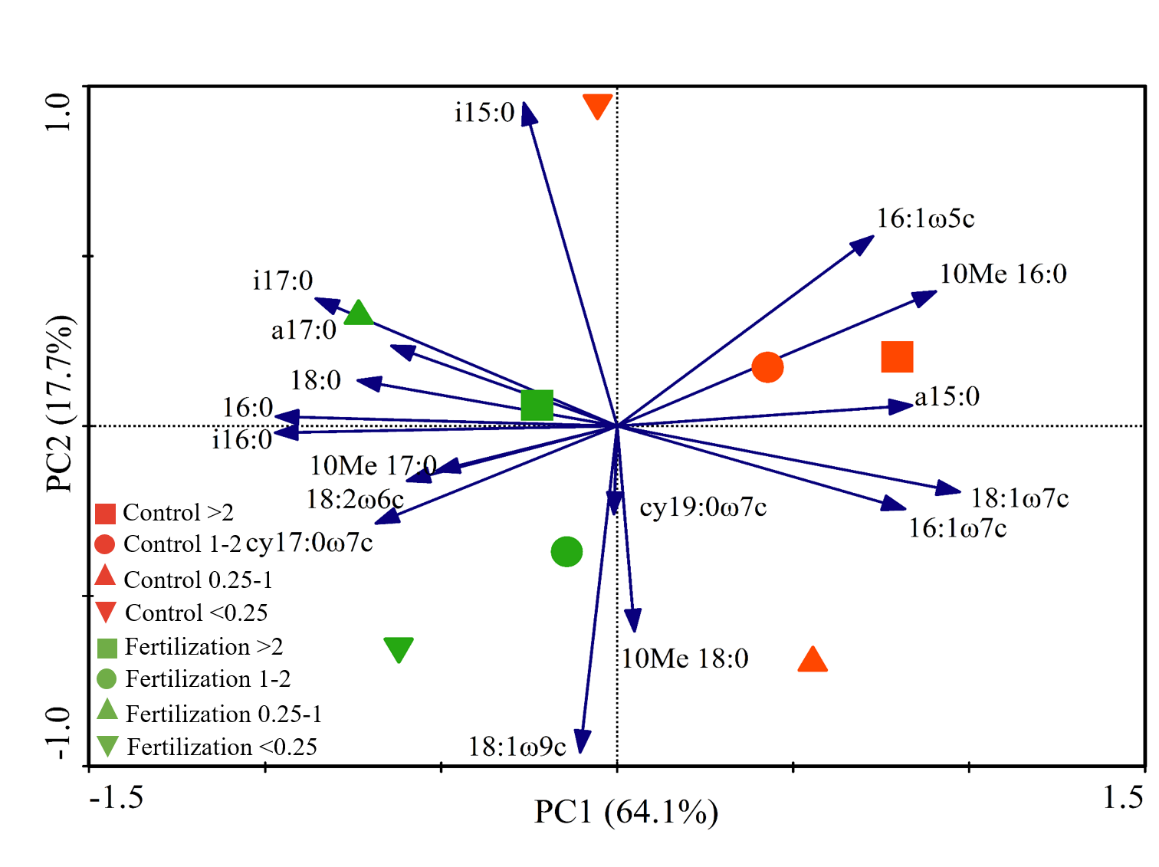


**Fig. S1** Principle component analysis (PCA) based on the relative proportion of individual PLFA.

**

**

**Fig. S2** The MurA/bacterial PLFA (a) and GluN/fungal PLFA (b) ratios in bulk soil and different aggregate fractions. Error bars indicate standard deviations. Different lowercase letters mean significant difference (*P* < 0.05) among different soil aggregate fractions in the same fertilization treatment, and different uppercase letters mean significant difference (*P* < 0.05) between fertilization and no fertilization treatments in the same aggregate fraction.
